# Widening Access: Sterile Tourniquets for Surgery to the Distal Humerus

**DOI:** 10.7759/cureus.46148

**Published:** 2023-09-28

**Authors:** Christian Warner, Christopher Peach, Ronnie Davies

**Affiliations:** 1 Trauma and Orthopaedics, Manchester Shoulder and Elbow Unit, Wythenshawe Hospital, Manchester University Hospitals NHS Foundation Trust, Manchester, GBR

**Keywords:** mid shaft humerus fracture, humerus shaft fractures, upper limb surgery, upper extremity trauma, midshaft humerus fractures, elastic exsanguination tourniquet, sterile tourniquet, distal humerus fracture, tourniquet use

## Abstract

Purpose of the study

The use of tourniquets during surgery of the distal humerus can improve visibility and reduce surgical time. However, the available operating field can be limited due to the size and placement of the tourniquet. This proof-of-concept study aimed to determine if sterile tourniquets can provide a wider surgical field compared to non-sterile tourniquets for procedures around the distal humerus.

Methods

Volunteers (n = 5) were positioned to simulate access to the distal humerus. The distance from the posterior corner of the acromion to the tip of the olecranon was measured. Participants were draped according to the standard protocol for the use of a non-sterile or sterile tourniquet for distal humerus and humeral shaft fractures. Two non-sterile pneumatic tourniquets (standard and narrow) and two sterile tourniquets (pneumatic and elastic exsanguination) were tested. The surgical field was measured from the sterile drape or tourniquet proximally to the tip of the olecranon. A one-way repeated measures ANOVA was conducted to examine the effect of each tourniquet on the surgical field.

Results

The sterile elastic exsanguination tourniquet had the largest available field with a mean of 24.4 cm (71% of arm available for incision after application), followed by the sterile pneumatic tourniquet of 20.0 cm (58%), narrow non-sterile pneumatic of 19.2 cm (55%), and standard non-sterile pneumatic of 17.0 cm (49%). Repeated measures ANOVA determined that mean surgical field length is statistically significant between tourniquet devices (F (1.729, 6.914) = 21.783, p = .001). The surgical field length was statistically significantly increased from a non-sterile standard tourniquet to a sterile elastic tourniquet (7.4 (95% CI, 2.9-11.9) cm, p = .008) but not the other two tourniquet devices tested.

Conclusion

The use of certain types of sterile tourniquets can provide a wider surgical field compared to non-sterile tourniquets for procedures around the distal humerus, specifically the sterile elastic exsanguination tourniquet providing a statistically significant mean gain of 7.4 cm from the non-sterile tourniquets. These findings suggest that the use of sterile tourniquets should be considered more frequently in surgery of the distal humerus, and a sterile exsanguinating tourniquet could be considered for midshaft humeral fractures, facilitating safer exposure of the radial nerve and reduced blood loss.

## Introduction

Since Lister in 1864, the elastic “Esmarch” bandage in 1873, and the initial use of the pneumatic tourniquet in 1904 by Harvey Cushing, tourniquets have been used for limb surgery. Being introduced with the intention to create a bloodless surgical field, tourniquets have become commonplace in orthopaedic and limb surgery [1-9]. The use of a tourniquet in limb surgery makes operations easier and faster as well as improves identification of important structures [5]. Guidance on intra-operative limb tourniquets within the United Kingdom is published and updated by the British Orthopaedic Association Standards for Trauma and Orthopaedics (BOASTs) to guide UK operative practice [10].

Pneumatic tourniquets are available as either non-sterile or sterile devices. The use of a non-sterile tourniquet is more common when compared to the use of sterile devices because of its multi-patient use and overall lower cost [4,11]. Increasing the use of sterile tourniquets has been recommended based on research suggesting that re-useable inflatable limb tourniquets pose a potential infection risk that could lead to significant complications and also have an associated cost attributed to these complications [7,12,13]. Re-useable tourniquets are commonly used far in excess of the manufacturing recommendations' maximum number of uses and are often cleaned improperly or infrequently [4,6,13]. The colonisation of re-useable (non-sterile) pneumatic tourniquets has been found to be 68%-78%, with a variety of microorganisms present, including skin flora and those common in post-operative infections [4,13].

Even before the data suggesting the colonisation of non-sterile tourniquets, surgical practice dictated that a non-sterile tourniquet must be excluded from the surgical field to prevent contamination. As a result, surgical drapes are positioned distal to the tourniquet to facilitate a safe barrier between the device and the operating field [2,6]. This inherently impacts the size of the surgical field as any incision must be made distal to the edge of the surgical drape to prevent contamination. However, the sterile pneumatic tourniquet can be considered as part of the sterile surgical equipment and is applied after the limb is decontaminated and draped. The use of a sterile tourniquet changes the placement of sterile surgical drapes, allowing for the tourniquet to reside within the sterile surgical field and incisions to be made up to the edge of the device [7].

The aim of this proof-of-concept study was to explore whether this difference in draping and tourniquet placement results in a larger and more accessible surgical field for upper limb orthopaedic surgery, primarily with its relevance to intervention for the distal humerus. Conventionally, the use of an intra-operative tourniquet is more limited to orthopaedic procedures on or surrounding the distal 1/3 of the humerus due to the size of the sterile surgical field after draping. This limits the use of a tourniquet for fractures that extend more proximally to the shaft and for various other indications of the midshaft and more proximal regions.

To account for the variety of different limb tourniquets available in the market for use in upper limb surgery, we tested those that were most readily and easily available: two non-sterile pneumatic tourniquets (standard and narrow) and two sterile tourniquets (pneumatic and elastic exsanguination). The sterile elastic tourniquet known as HemaClear (OHK Medical Devices, Haifa, Israel) is a silicone ring rolled within a stockinette. This is unrolled onto the limb to the desired occlusion site providing simultaneous limb exsanguination and supra-systolic tourniquet effect [1,2,6,11,14]. There are a variety of sizes equating to limb circumference available for upper and lower limbs. Although not formally quantified, the use of the sterile elastic tourniquet has been described to improve the size of the surgical field for limb operations [7,15].

## Materials and methods

**Figure 1:**
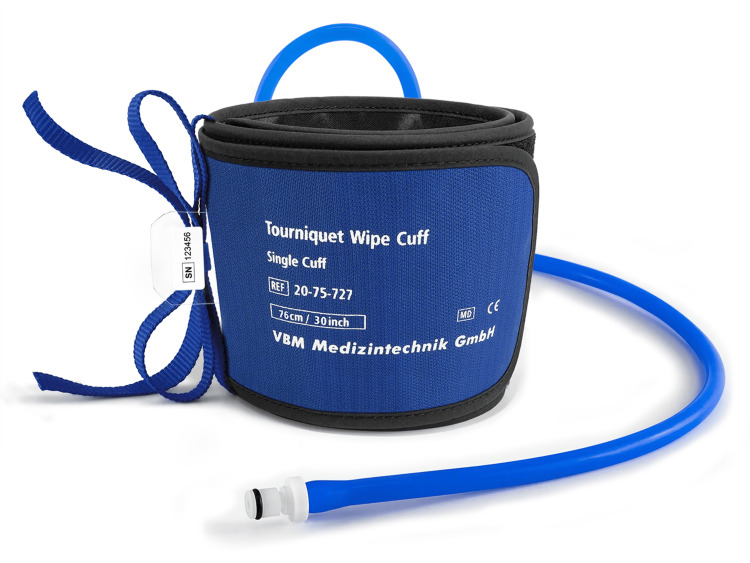
Example of non-sterile standard pneumatic tourniquet (NST) Image rights are attributed to VBM Medizintechnik GmbH, and permission has been obtained for using the image.

**Figure 2:**
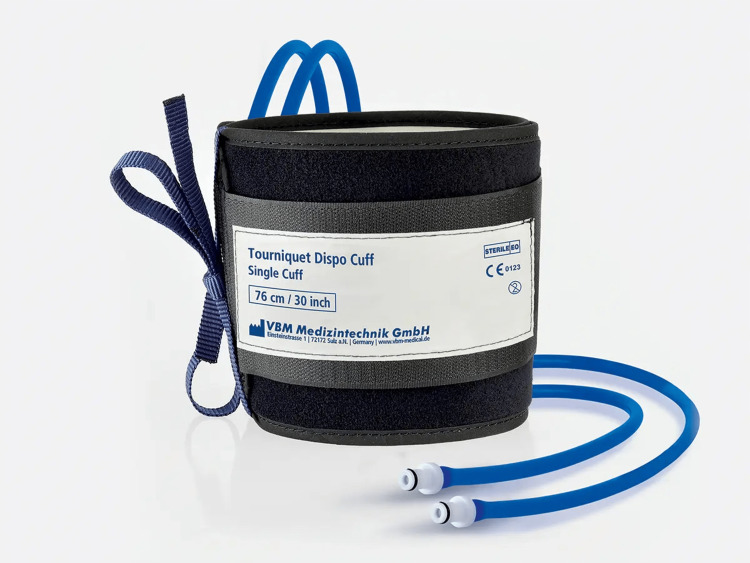
Example of sterile standard (pneumatic) tourniquet (SST) Image rights are attributed to VBM Medizintechnik GmbH, and permission has been obtained for using the image.

**Figure 3:**
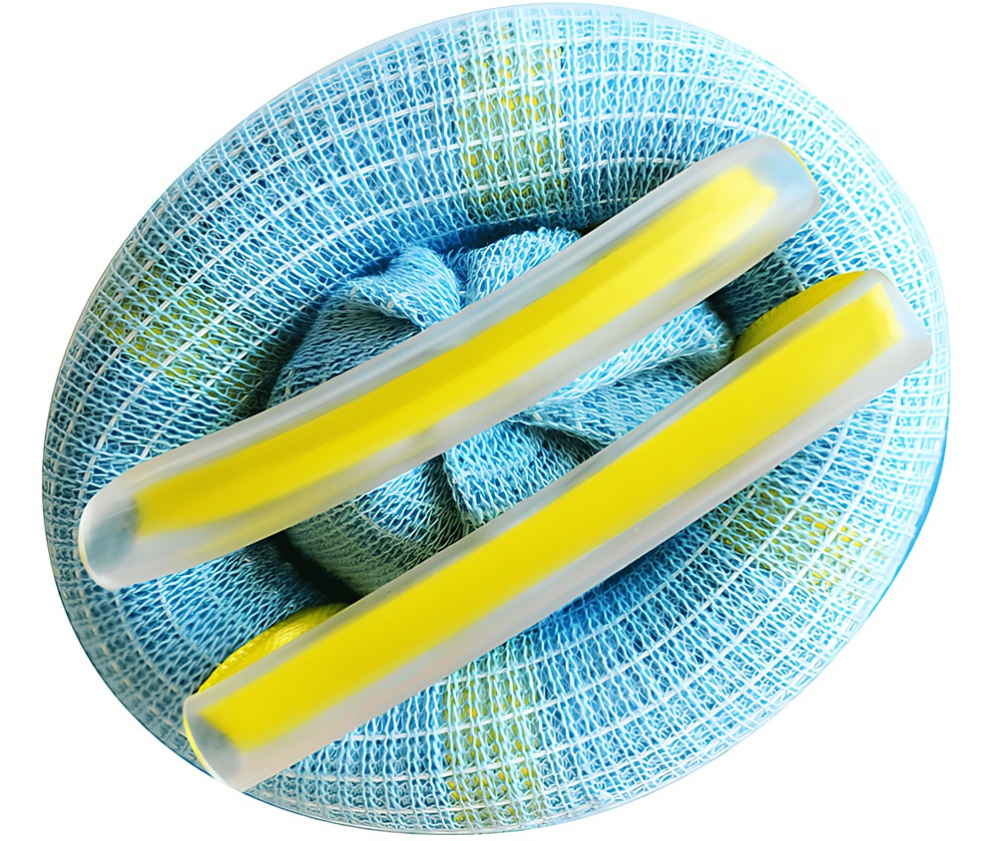
HemaClear sterile elastic (exsanguination) tourniquet Image rights are attributed to VBM Medizintechnik GmbH, and permission has been obtained for using the image.

The tourniquets in use were as follows: (1) non-sterile standard pneumatic (NST), as shown in Figure 1; (2) non-sterile narrow pneumatic (NNT); (3) sterile standard pneumatic (SST), as shown in Figure 2; and (4) sterile elastic exsanguination (SET), as shown in Figure 3. All four devices were current stock and in use within the department based on individual preference and practice. Each tourniquet has a slight variation in the measured width by device: NST 110 mm, NNT 90 mm, SST 104 mm, and SET 14 mm. The SET was standard upper limb medium (yellow) suitable for arms of circumference 24-40 cm.

As per the NHS Health Research Authority guidance, this study did not require NHS Research Ethics Committee (REC) review for sites in England. We recruited five volunteers from the hospital orthopaedic department on an opportunistic basis, aiming to assess a mix of sex, height, and body habitus. Informed consent for the study was obtained verbally for each participant. The length of the participants’ left arm was measured from the posterior acromion to the tip of the olecranon, with the elbow flexed to 90 degrees, by two members of the research team independently, and a mean was taken of the two values for any discrepancy. The maximum upper arm circumference was also measured using the same protocol.

**Figure 4:**
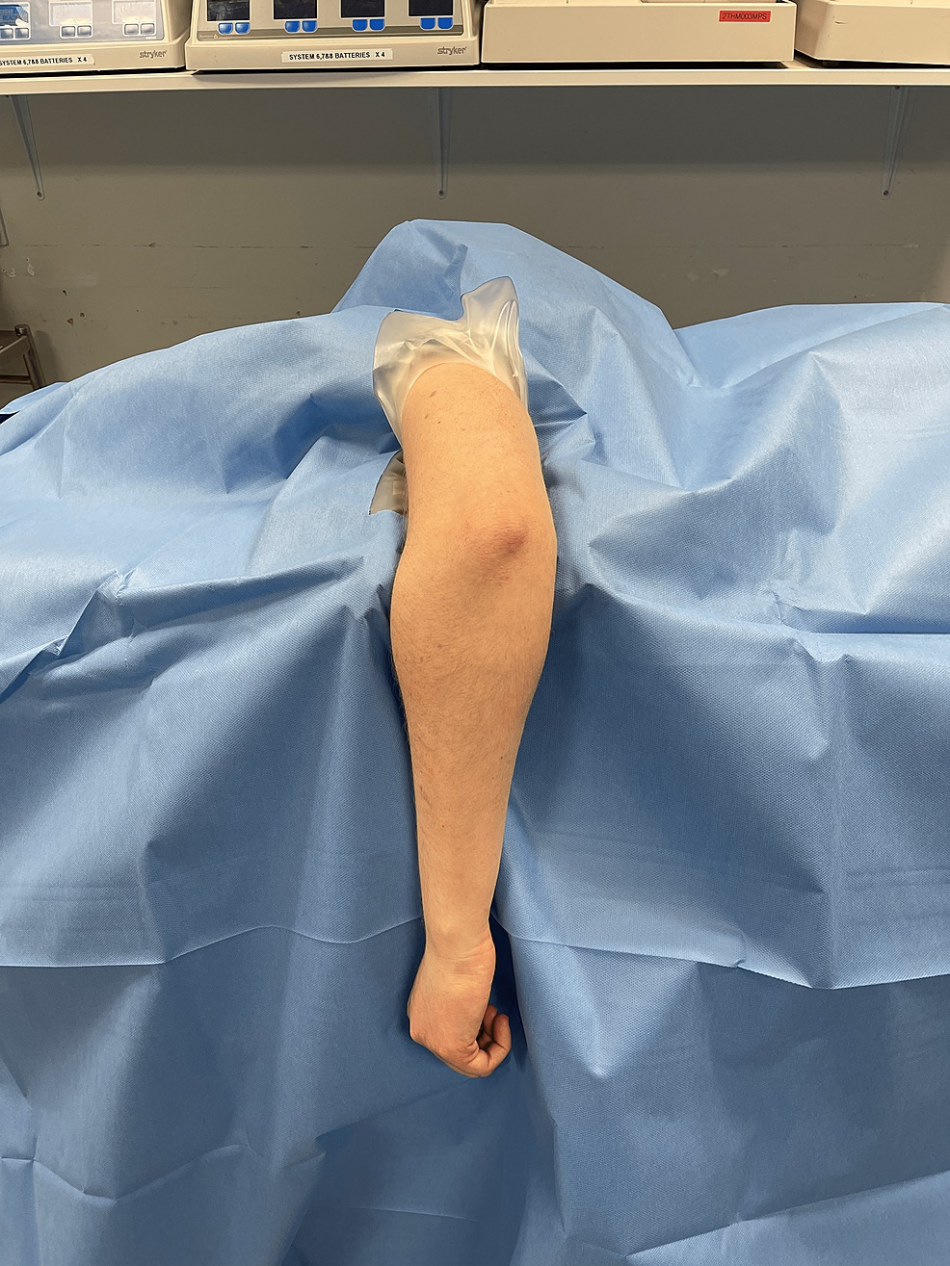
Positioning and draping in the right lateral decubitus

**Figure 5:**
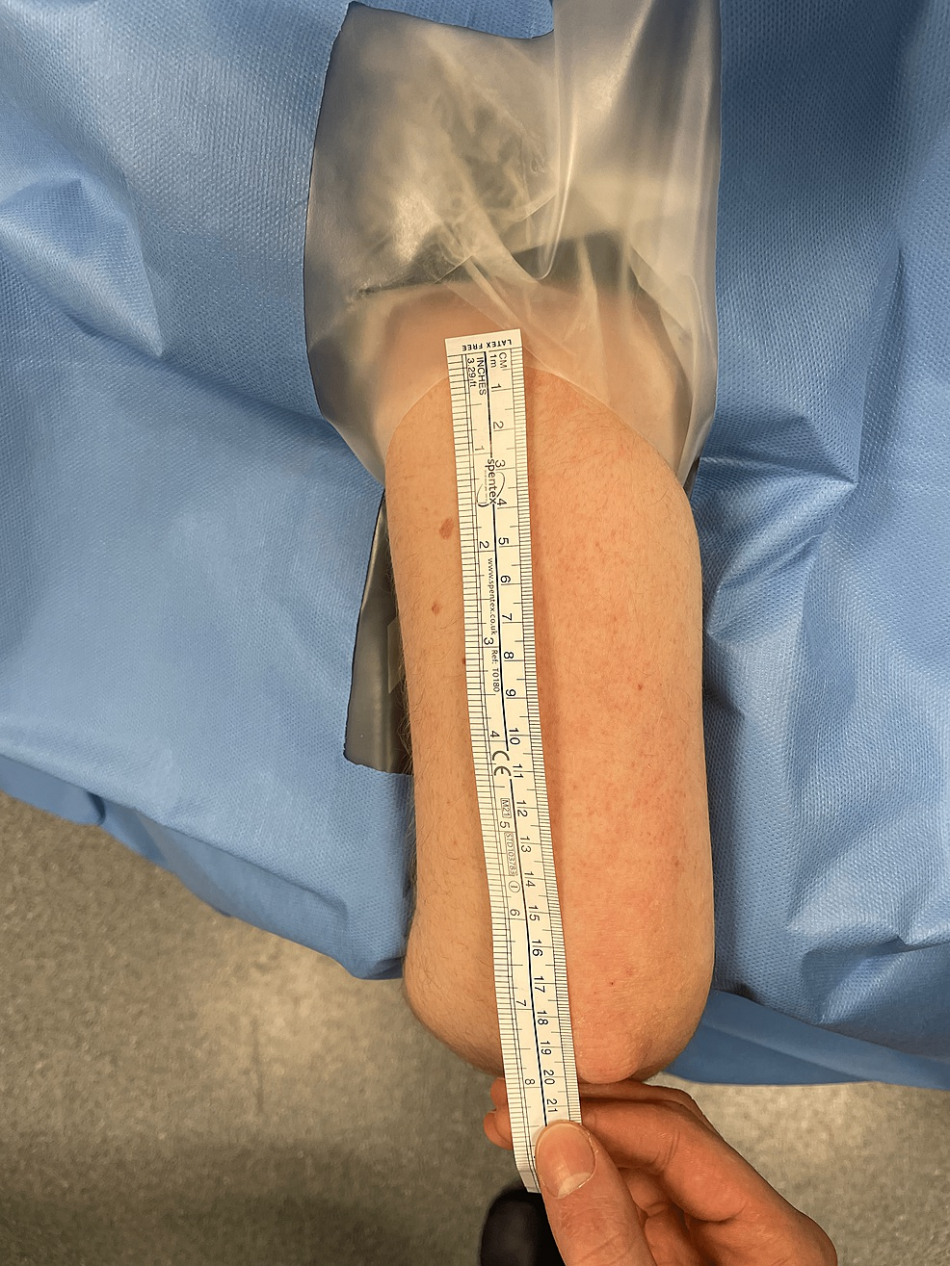
Measurement of the non-sterile standard tourniquet (NST)

**Figure 6:**
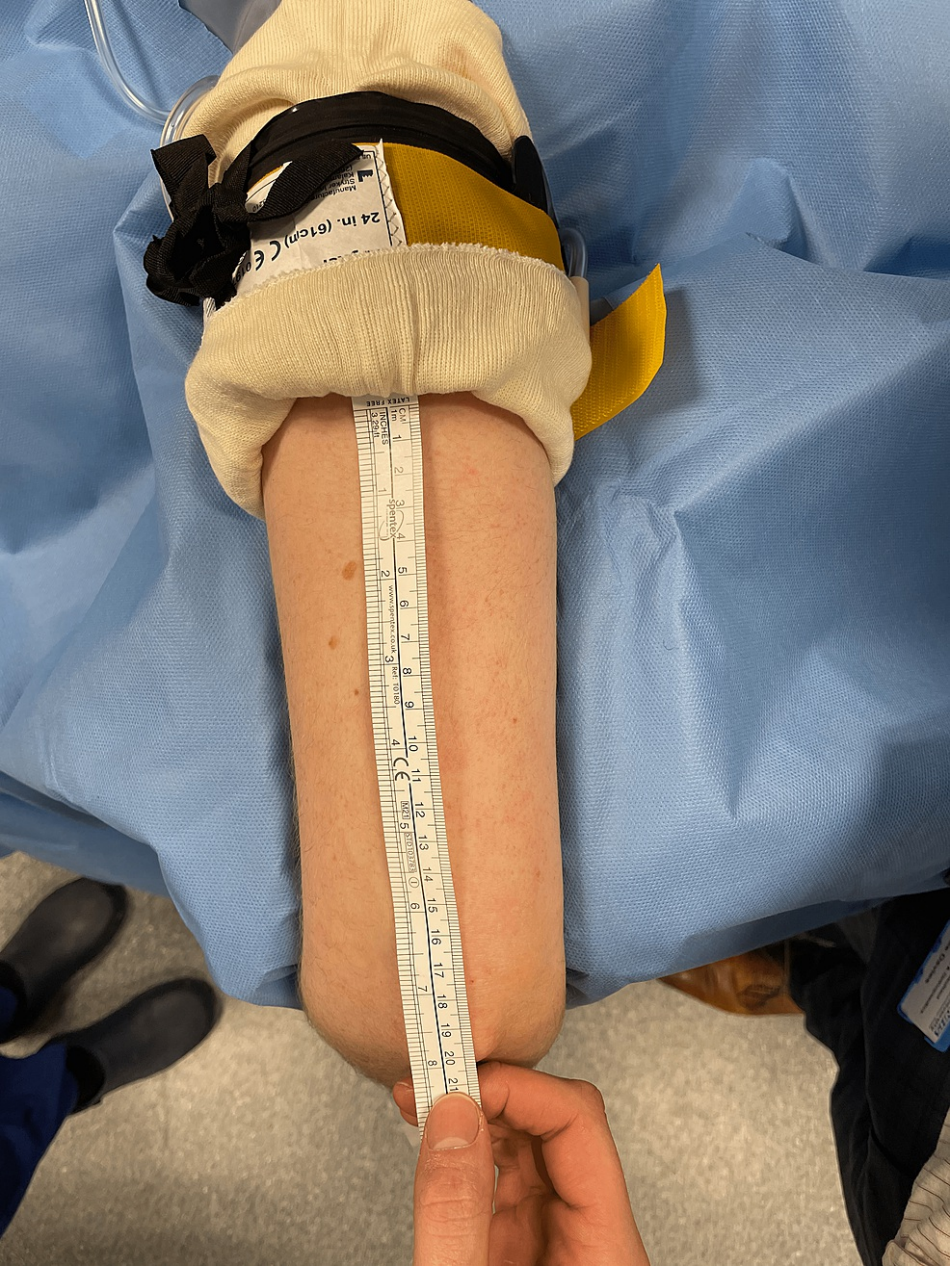
Measurement of the sterile standard tourniquet (SST)

**Figure 7:**
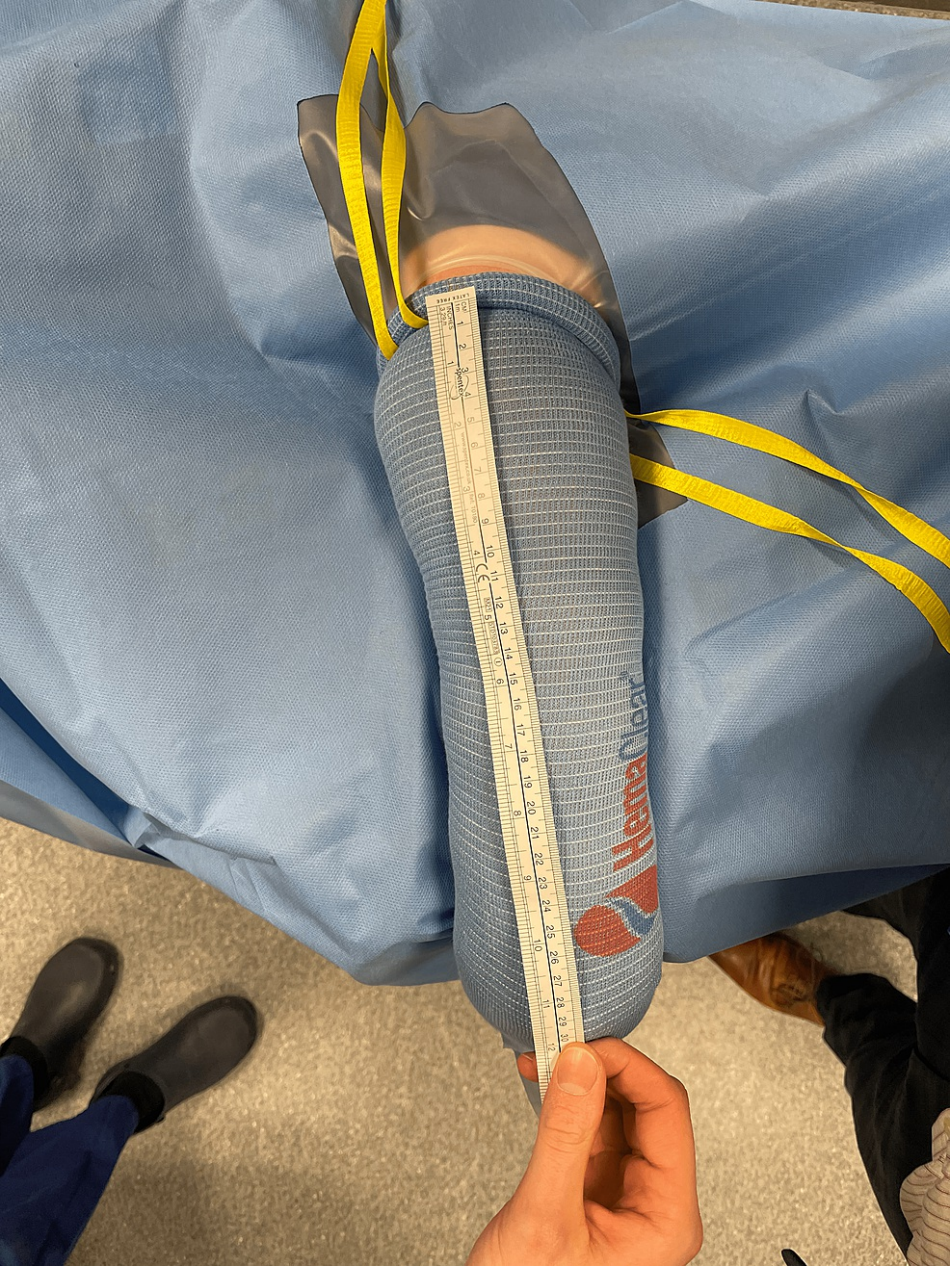
Measurement of the sterile exsanguination tourniquet

Participants were positioned in the right lateral position on a clean operating table within the operating theatre complex and draped according to the standard department practice, using an orthopaedic upper limb extremity drape with an elastic limb opening and the arm placed over an arm bar (Figure 4). A variation in draping was only altered for sterile versus non-sterile tourniquets to allow for the inclusion of the sterile tourniquet within the sterile surgical field. The upper limb extremity drape was positioned as proximal as possible without (1) including the axilla in the sterile tourniquet group, (2) exposing any of the non-sterile tourniquets, or (3) causing any gaping of the elasticated opening. The tourniquets were applied using standard protocols with padding beneath and inflated for measurement and then promptly deflated and/or removed to minimise discomfort. Measurement of the length of the useable surgical field was taken from the most proximal part of the sterile field, either up to the surgical drape or the sterile tourniquet, along the posterior aspect of the arm to the tip of the olecranon (Figures 5-7).

IBM SPSS Statistics for Macintosh, version 29.0 (IBM Corp., Armonk, NY) was used for statistical testing, with significance defined as a p-value < 0.05. Significance testing was performed using ANOVA with repeated measures when making comparisons between the operative field spaces available for each of the devices.

## Results

Of the five participants, three were female and two were male. The arm length and circumference of each participant are displayed in Table 1. The average arm length was 34.8 cm, and the average sterile surgical field length with the NST was 17 cm (12.5-20.5 cm), with NNT of 19.2 cm (17.5-21.5 cm), SST of 20.0 cm (17-21.7 cm), and SET of 24.4 cm (21.5-30.5 cm) (Table 2).

**Table 1:** Participants' sex and arm dimensions

Participants	Sex	Arm length (cm)	Arm circumference (cm)
1	Female	33.2	27.5
2	Male	37.1	35
3	Female	34.5	38.9
4	Female	33.5	28
5	Male	35.5	32.5

**Table 2:** Sterile surgical field length NST: Non-sterile standard pneumatic; NNT: Non-sterile narrow pneumatic; SST: Sterile standard pneumatic; SET: Sterile elastic exsanguination.

Participant	NST (cm)	NNT (cm)	SST (cm)	SET (cm)
1	20.5	21.5	21	30.5
2	18.5	20	21.7	23.5
3	12.5	17.5	17	21.5
4	17	18	21	24
5	16.5	19	19.5	22.5
Mean	17	19.2	20	24.4
Standard deviation	2.96	1.60	1.88	3.54

A repeated measures ANOVA with a Greenhouse-Geisser correction determined that mean surgical field length differed statistically significantly between tourniquet devices (F (1.729, 6.914) = 21.783, p = .001). Post-hoc analysis with a Bonferroni adjustment revealed that surgical field length statistically significantly increased from NST to sterile elastic tourniquet (7.4 (95% CI, 2.9-11.9) cm, p = .008) but not from NST to non-sterile narrow tourniquet (2.2 (95% CI, -1.4-5.8) cm, p = .258) or from NST to SST (3.0 (95% CI, -0.3-6.4) cm, p = .07).

## Discussion

The results demonstrate that the use of sterile tourniquets can increase the surgical field length compared to non-sterile tourniquets during procedures on the humerus. The SET had the largest available field with a mean of 24.4 cm (71% of arm available for incision after application), followed by the SST 20.0 cm (58%), NNT 19.2 cm (55%), and standard non-sterile pneumatic 17.0 cm (49%). This equates to a statistically significant (p = .008) increase in the mean surgical field of 7.4 cm (22% of total arm length) between the SET and NST. In comparison between the SST and NST, there was a trend towards an increased surgical field, but it did not reach statistical significance, most likely due to the sample size recorded.

This adds to the current literature as there is currently no published data with the primary goal of assessing the difference in the size of the surgical field between sterile and non-sterile (re-useable) pneumatic tourniquet devices in the upper limb. Our data shows that the SET had the largest surgical field, which is logical given that it is also the narrowest device (14 mm), compared to the widest NST (110 mm). However, the SST showed an increase of 3 cm in the surgical field (9% of total arm length) despite its width of 104 mm being only 6 mm narrower than the NST. Therefore, it is likely that some of the attributable increases in the surgical field with alternate devices to the NST may relate to the width of the tourniquet. However, reducing the width has restrictions, such as the potential risk of increased pressure over a smaller area.

During knee arthroplasty, the use of a sterile elastic tourniquet increased the average operative field length to 14.4 cm compared to a standard non-sterile pneumatic tourniquet of 5.5 cm, with a difference of 8.9 cm. The increased size of the operative field in knee arthroplasty was linked to reduced surgical time and complications. It is believed to reduce the risk of contamination by reducing the need to extend incisions close to the edges of the sterile field while increasing sterile exposure [4]. It has also been found that the use of the SET in total knee arthroplasty was associated with reduced intra-operative bleeding and post-operative bleeding into drains, a smaller fall in haemoglobin, and a lower rate of wound infection postoperatively [4,16,17].

Three studies reported that participants experienced less pain and soft tissue reaction with the use of a sterile elastic tourniquet to the upper arm, compared to a pneumatic tourniquet, along with a reduced incidence of paraesthesia postoperatively [1,17,18]. The use of the narrower sterile silicone elastic tourniquet has also been reported to provide an improved fit for patients with tapered or conical-shaped limbs, particularly paediatric patients, in which traditional pneumatic tourniquets were more liable to slip distally, reducing the effectiveness of arterial occlusion and impeding sterility. This has facilitated the use of a tourniquet in procedures where it would otherwise not have been possible to do so, with the benefit of a bloodless field [7,11]. We found that the SET did fit differing arm shapes, but it rolled back to a certain extent on those with more conical-shaped arms in the awakened state from its most proximal position.

A published case report has raised caution about the use of a SET in trauma cases or in patients with high-risk deep vein thrombosis (DVT) in the affected limb, following two cases of pulmonary embolisms after SET application [19].Both cases related to fractures of the lower limb, and it is postulated that the exsanguination process in the application of the SET may have dislodged an existing DVT. It is unclear whether these DVTs could have been mitigated, and the evidence given in only two cases is weak, but it is recommended to use caution and consideration for cases with high risk of DVT such as delay following injury.

Distal humerus fractures account for 2%-3% of all adult fractures, with the incidence expected to increase in an ageing population. The demographics are typically that of younger adults involved in trauma and those aged 65 and above who sustain low-energy falls [20-22]. Open reduction internal fixation is the mainstay of treatment for most distal humerus fractures, depending on the function of the patient [20,21].

Complex distal humerus fractures may extend more proximally beyond the metaphysis into the diaphyseal region. A tourniquet device that allows more proximal access therefore would provide an advantage, potentially making it possible to use a tourniquet where a non-sterile tourniquet could not be used. Standard pneumatic tourniquet devices with sterile draping often limit the surgical field to the distal 1/3 of the upper arm [12]. Our results suggest that an NNT, SST, and SET would all allow greater proximal access, particularly the SET with greater than 70% of the arm length within the sterile operating field. More complex distal humerus operations take longer and, as a result, have a higher rate of complications such as infection. The use of a tourniquet to create a bloodless field is associated with a reduced duration of surgery and the identification of important structures, which are both likely to contribute to improved outcomes where a tourniquet can be used [5].

A significant limitation of this analysis is the small number of participants (n = 5) in the study group, who were opportunistically selected. Despite opting to have a mixture of sexed participants, this selection method increases the risk of selection bias. The sample size is underpowered for picking up smaller differences between surgical field sizes. Any more robust data set should be larger and more systematically selected, which reflects patients undergoing distal humerus operations. The applicability of these initial results is limited as it has not quantified the size of the surgical field required for operative approaches on a case basis. Instead, this will continue to be a surgeon-led decision as to whether the increased surgical field size of using a sterile tourniquet allows proximal enough access for each specific case.

## Conclusions

In conclusion, the use of certain types of sterile tourniquets can provide a wider surgical field than non-sterile tourniquets during procedures around the distal humerus. More specifically, the sterile elastic exsanguination tourniquet demonstrated the largest available field with a statistically significant difference from the non-sterile tourniquets. The results of this proof-of-concept study suggest that the use of sterile tourniquets should be considered more frequently in the surgery of the distal humerus. The placement of the sterile tourniquet within the sterile surgical field allows for incisions to be made up to the edge of the device, resulting in a larger and more accessible surgical field. These findings may have implications for orthopaedic surgical procedures beyond those focused on the distal humerus such as midshaft humeral fractures, facilitating safer exposure of the radial nerve and reduced blood loss.
